# Research Progress on the Utilization of Semi-Dry Calcium-Based Desulfurization Dross in China

**DOI:** 10.3390/ma18194455

**Published:** 2025-09-24

**Authors:** Min Pan, Ruiying Wang, Shejiao Yan, Xiangqian Du, Zhenxing Yin, Guangchao Wu, Jiamao Li, Canhua Li

**Affiliations:** 1 China Metallurgical Huatian Engineering Technology Co., Ltd., Ma’anshan 243002, China13965396242@163.com (S.Y.); duxiangqian666@163.com (X.D.); 2 School of Metallurgical Engineering, Anhui University of Technology, Ma’anshan 243002, Chinaw860063878@163.com (G.W.); 3 School of Materials Science and Engineering, Anhui University of Technology, Ma’anshan 243002, China; agdjiamaolee@126.com

**Keywords:** semi-dry process, calcium-based desulfurization dross, resource utilization, harmless disposal

## Abstract

As a solid waste generated during the desulfurization process of coal-fired power plants, the output of desulfurization dross is increasing year by year. If not properly treated, it may occupy land and potentially pollute the environment. This article reviews the physicochemical properties of desulfurization dross and the progress in its resource utilization. It specifically focuses on the application potential of semi-dry desulfurization dross, emphasizing how its comprehensive resource utilization can reduce environmental pollution and generate considerable economic benefits for related industries. It should be noted, however, that the leaching of heavy metals and the strong alkalinity of desulfurization dross may pose environmental risks such as soil and groundwater contamination. Current research still requires further improvement in the systematic assessment and management strategies of these risks. This review highlights the need to optimize pretreatment technologies for stabilizing desulfurization dross and enhance environmental risk management, to facilitate its large-scale and high-value utilization. This article also looks toward the research directions for semi-dry calcium-based desulfurization dross in the future, aiming to provide a reference for the sustainable development and environmental protection of semi-dry desulfurization dross.

## 1. Introduction

With the continuous intensification of the global warming trend, climate change has become a hot issue of focus for all countries in the 21st century. Currently, we are facing severe challenges posed by climate change, and we also need to address the serious harm caused by air pollution [1,2]. Sulfur dioxide (SO_2_) belongs to the category of atmospheric pollutants; it is a colorless, pungent-odorous, toxic gas, which can have a significant impact on human health and the atmospheric environment [3]. The Green Environment Trust (GET) reported in its 2020 report that China was the third-largest emitter of SO_2_ in the world, accounting for 8% of the global total of anthropogenic SO_2_ emissions [4]. The 2021 China Environmental Status Report, which was released, showed that 98.2% of the cities in China had reached the national-level standard for sulfur dioxide, and the area of acid rain regions accounted for 3.8% of the total national land area [5]. McLinden et al. [6] identified previously unreported sulfur dioxide emission sources via satellite observations, which are predominantly located in developing countries and account for approximately 6–12% of global anthropogenic emissions. Acid rain in China is mostly of the sulfurous type, which mainly originates from the large emissions of SO_2_. The hazards of acid rain are widespread, and it will damage soil, vegetation, human health, buildings, etc. The emission problem of SO_2_ cannot be ignored [7]. When SO_2_ is emitted into the atmosphere, it is oxidized by air to form sulfate particles, which can adsorb water vapor, thus forming acid rain, causing multi-faceted harm to the ecological environment. Firstly, acid rain accelerates the erosion rate of the vegetation surface, destroys the tissue structure and chlorophyll of leaves, and thus inhibits the photosynthesis of plants, affecting their normal growth and development [8]; acid rain also causes damage to microorganisms in the soil, making the interaction between them more frequent, thus not only inhibiting their cooperative behavior but also intensifying their competition [9]; acid rain caused by industrial emissions, when it falls into rivers, lakes, and other waters, will cause water acidification, which will increase the solubility of some heavy metal ions, and this has a great impact on the content of heavy metals in aquatic sediments, thus poisoning aquatic organisms and destroying the balance of the aquatic ecosystem [10]; the sulfate ions in acid rain will degrade cement hydration products in concrete, and acid rain through roads, buildings and other structures will produce corrosive effects, accelerating aging and shortening their service life [11,12]; in addition, SO_2_ also poses serious hazards to humans, as long-term exposure to sulfur dioxide can irritate the respiratory system and also increase the risk of cancer.

According to the National Bureau of Statistics’ “Statistical Bulletin on National Economic and Social Development in 2021”, the steel production in China in 2021 was 1.337 billion tons [13]. The steel industry, as a high energy-consuming and high-emission industry, released a large amount of SO_2_ in the production process, and the emitted SO_2_ accounted for more than 30% of the total industrial emissions, becoming one of the main sources of SO_2_ emissions in China. With the continuous and rapid development of China’s economy, the extensive application of new technologies in the industrial field had significantly improved the production efficiency. In recent years, along with the continuous improvement of national requirements for environmental protection, flue gas desulfurization technology has developed rapidly, and the integrated and collaborative treatment of desulfurization, nitrification, and dust removal has become the mainstream management model for iron and steel enterprises. The Law of the People’s Republic of China on the Prevention and Control of Environmental Pollution by Solid Waste explicitly stipulates that industrial waste management must adhere to the principles of reduction, resource recovery, and harmless treatment; generating units are required to establish a whole-process pollution prevention responsibility system to minimize waste generation at the source [14]. Among various desulfurization technologies, semi-dry desulfurization is widely used with a relatively low investment cost, small footprint, dry by-products from desulfurization. [15,16]. According to statistics, the output of desulfurization by-products in China reached 11 million tons in 2020, and that of dry desulfurization dross exceeded 30 million tons [17]. The steel industry emits about 20 million tons of semi-dry desulfurization dross every year nationwide, and semi-dry desulfurization dross, as a by-product of desulfurization after processing, has become a bulk solid waste, urgently needing mature and reliable treatment technology in order to achieve the comprehensive utilization of semi-dry desulfurization dross [18]. Semi-dry flue gas desulfurization technology mainly includes the circulating fluidized bed method (CFB) and spray-drying absorption (SDA) [19]. The CFB system uses powdery Ca(OH)_2_ as the absorbent, and the flue gas and absorbent are mixed and reacted in a tower. The temperature is controlled by adjusting the amount of water sprayed, no wastewater is produced, the adaptability is strong, the load adjustment range is wide, the desulfurization dross is recycled, and the desulfurization efficiency can reach more than 98%. SDA sprays the lime slurry into the absorption tower to make it contact and react with the flue gas. However, the process has some disadvantages: core equipment—the rotating atomizer is prone to wear, resulting in high maintenance costs; the system is prone to scaling and blockage problems, and the digested sludge is to be discharged outside; moreover, the process has relatively poor adaptability to changes in flue gas load and SO_2_ concentration; and the desulfurization efficiency is usually less than 90%. The flue gas circulating fluidized bed desulfurization process (CFB-FGD) has become one of the best semi-dry desulfurization processes, with a comprehensive performance after more than thirty years of continuous improvement [20]. However, the Ca/S ratio of the semi-dry process is higher than that of the process, which will lead to an increase in the production of desulfurization dross with low utilization, resulting in more difficult treatment of desulfurization dross, which there is a need to study [21].

Harmful gases such as sulfur dioxide emitted by industries have become an important source of atmospheric pollution. With the promotion of national environmental protection policies, a large number of enterprises have introduced sintering flue gas desulfurization devices, which have not only effectively reduced pollutant emissions but also brought about new problems that urgently need to be solved. At present, sulfur dioxide’s main disposal methods are abandonment and stacking; however, such practices will not only cause secondary pollution to water and soil but also severely damage the atmospheric environment [22]. During the natural leaching of desulfurization dross, the leached mass concentrations of heavy metals such as Pb, Ni, and Cd exceeded the limits set by the West German landfill standards for solid waste by 0.4 to 1.47 times [23]. If disposal sites lack protective measures such as impermeable layers and rain shelters, leachate can mobilize heavy metals from the dross, compromising the safety of its subsequent resource utilization. The environmental risks of semi-dry desulfurization dross represent a core challenge that must be addressed in its resource recovery, primarily due to its complex physicochemical properties. First, the risk of heavy metal pollution is particularly prominent. The desulfurization process effectively captures and enriches trace heavy metal elements from flue gas. Studies indicate that desulfurization dross from iron and steel sintering processes is a key carrier for Tl enrichment, far exceeding levels commonly found in general industrial solid waste [24]. In addition, various heavy metals such as As, Cr, lead Pb, and Zn are frequently detected [25]. Under natural storage conditions, these toxic elements are highly prone to leaching and migration through rainwater, posing long-term and hidden threats to surrounding soil and groundwater ecosystems, and ultimately endangering human health through the food chain. Second, desulfurization dross typically contains large amounts of unreacted absorbents, such as CaO and Ca(OH)_2_, rendering it highly alkaline, along with an elevated Cl^−^ content [26]. These properties can alter the pH of adjacent soil upon direct exposure, disrupt the soil aggregate structure and microbial community balance, and potentially exacerbate soil salinization. In early wet desulfurization processes, wastewater enriched with these pollutants exhibited thallium concentrations more than ten times above regulatory standards, highlighting its significant potential for environmental mobility [24].

Therefore, the safe disposal and resource utilization of desulfurization dross are not merely considerations for economic benefit but essential measures to block the pathways of pollutant diffusion and uphold the principle of environmental risk prevention. Although semi-dry desulfurization dross had been preliminarily applied in the fields of building materials and agriculture due to its advantages such as cohesion and a high CaO content, there were still many problems: large component fluctuation leading to unstable product performance; incomplete oxidation of calcium sulfite affecting utilization safety; and an imperfect environmental risk system restricting large-scale application. This paper focuses on the resource utilization of semi-dry calcium-based desulfurization dross, firstly analyzing its physical and chemical properties, and identifying restrictive factors such as complex composition and poor stability; and then systematically sorting out the application research in the fields of building materials, agriculture, and wet flue gas desulfurization, analyzing the application potential in various fields. The aim is to provide a technical reference for the large-scale and high-value utilization of semi-dry desulfurization dross, and furthermore to outline its environmental and economic benefits.

## 2. Physical and Chemical Properties of Desulfurization Dross of Semi-Dry Method

Semi-dry desulfurization dross is a high-calcium and high-sulfur dross residue, and its composition is relatively complex, with a high content of CaSO_3_, low permeability, and unstable properties, which restrict the extensive application of desulfurization dross and are not conducive to the health of semi-dry desulfurization technology [18,27]. The desulfurization dross generated by the semi-dry desulfurization process is powder, and the composition of the desulfurization dross is significantly affected by the flue gas, meaning its color shows different states, such as reddish brown, light yellow, or grayish white. For instance, when treating sintering flue gas containing iron ore dust, the desulfurization dross absorbs approximately 1.57% Fe_2_O_3_ and 94.08% calcium sulfides, often resulting in a yellowish-white color. In cases where the iron content in the flue gas is lower, the dross contains only about 1.35% Fe_2_O_3_ and 41.25% calcium sulfides, typically appearing off-white. Further variations in iron content may lead to other colors, such as reddish-brown, directly correlated with the differing concentrations of iron compounds (e.g., Fe_2_O_3_) in the desulfurization dross depending on the flue gas source [17,19]. Over the past decades, various desulfurization processes have been developed by various industries, institutes, and universities, which have enabled the emission of pollutants in the flue gas of the iron and steel industry to meet the standard set, and which have contributed to the reduction in sulfur dioxide emissions and the reduction in the area of acid rain in China [28]. The type of desulfurizing agent directly determines the core composition of desulfurization dross, which can be classified as follows: calcium-based desulfurization dross, produced using calcium-based sorbents such as limestone, quicklime, or Ca(OH)_2_; magnesium-based desulfurization dross, generated with MgO or Mg(OH)_2_ as the sorbent; ammonia-based desulfurization dross, resulting from the use of ammonia water or liquid ammonia, primarily containing ammonium salts (e.g., ammonium sulfate and ammonium sulfite); and sodium-based desulfurization dross, formed with sodium carbonate (Na_2_CO_3_) or sodium hydroxide (NaOH) as the sorbent. Due to the high cost of sodium resources, its industrial application is relatively limited. Calcium-based desulfurization dross is the most common type, mainly composed of calcium sulfates/sulfites (such as CaSO_4_ and CaSO_3_). The classification basis of calcium-based desulfurization dross in the iron and steel industry is shown in Table 1.

### 2.1. Analysis of Particle Size of Desulfurization Dross Provided by a Company in Ma’anshan

**Figure 1:**
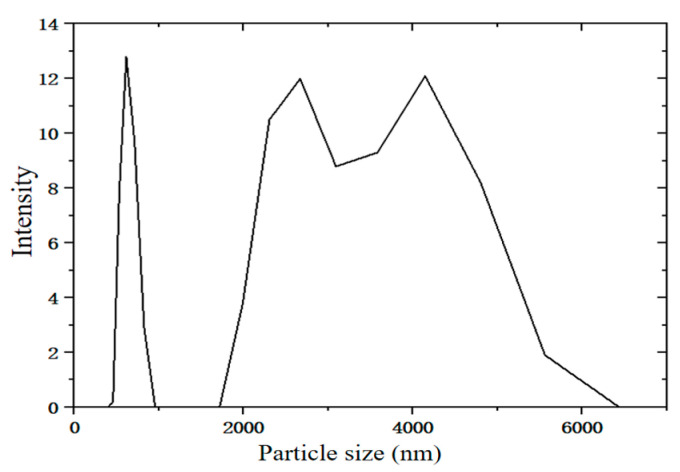
Nanoscale size distribution of desulfurization dross from a Ma’anshan company.

The particle size distribution of desulfurization dross can be analyzed to optimize its pretreatment processes (such as grinding), thereby improving its stability and applicability [29]. The desulfurization dross sample used in this test was provided by a certain company in Ma’anshan by the method of SDA. The particle sizes of the desulfurization dross were detected by the particle size analyzer (MalvernZetasizer Nano ZS90, produced in the Worcestershire, UK), and the results are shown in Figure 1.

It can be seen from Figure 1 that the average particle size of the desulfurization dross obtained by the SDA method was 2446 nm, with three peaks, that is, three groups of nanoparticles with different particle sizes, peaking at 641.1 nm, 2591 nm, and 3984 nm, respectively. Li et al. [30] detected the desulfurization dross produced by the SDA method at Anshan Iron and Steel, and the data showed that the median particle size (D50) of the desulfurization dross was 6460 nm, the volume average diameter was 8650 nm, and the area average diameter was 2960 nm. Qian et al. [19] detected the desulfurization dross obtained by the DFA method, and showed that 80% of the desulfurization dross particle sizes were above 1039 nm, while the median particle size (D50) was 4521 nm. It can be seen that the process of producing desulfurization dross by different enterprises was the same, and the law of particle size distribution was roughly the same, but the particle size of the desulfurization dross produced by the SDA method was significantly larger than that of the DFA method. The comparison between the SDA and DFA methods is shown in Table 2.

**Figure 2:**
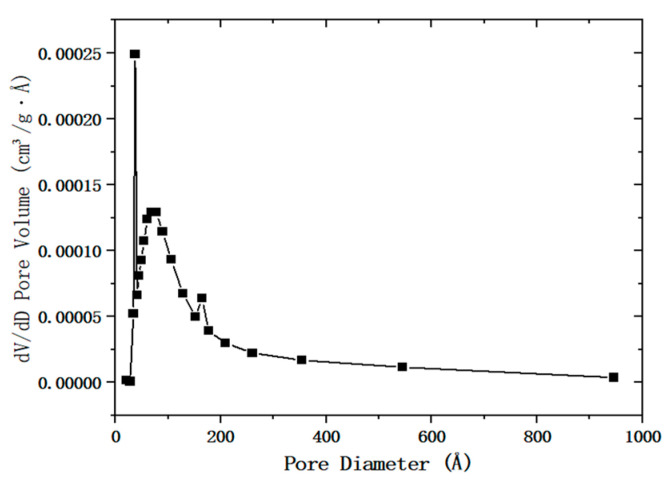
Distribution curve of desulfurization dross pore sizes from a Ma’anshan company.

**Figure 3:**
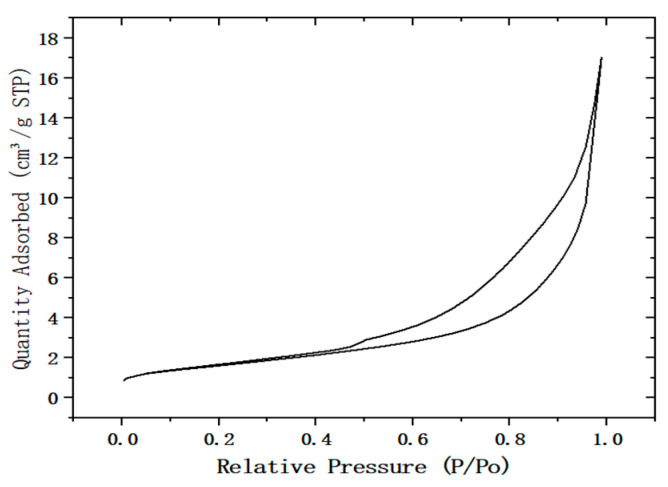
Adsorption and desorption curves of desulfurization dross from a Ma’anshan company.

The desulfurization dross was detected by the nitrogen adsorption–desorption instrument (BET), and the detection results of the pore size of the desulfurization dross are shown in Figure 2, while the adsorption and desorption curves of the desulfurization dross are shown in Figure 3.

Qian et al. [19] measured the specific surface area of the DFA method desulfurization dross as 2.25 m^2^/g. The measured results showed that the specific surface area of the SDA method desulfurization dross provided by a certain company in Ma’anshan was 5.868 m^2^/g, which was relatively large. The total pore volume of the desulfurized dross was 0.026282 cm^3^/g. The average pore diameter of the desulfurization dross was 13.1 nm, which belonged to mesopores. It can be seen from Figure 2 that most mesopores were located within 2-50 nm, and there were few pores larger than 50nm. The mesoporous structure endowed the desulfurization dross with a large specific surface area, and this property gave it potential in the field of adsorption, such as use as an adsorbent to remove heavy metal ions in wastewater treatment. However, the smaller pore size may also affect its performance in road base backfill, and it was necessary to optimize the pore structure by pretreatment (such as grinding) to improve the applicability [29].

From Figure 3, it can be seen that in the low-pressure area, the adsorption amount increased slowly, or may even have decreased slightly; in the high pressure area, the adsorption amount increased sharply with the increase in pressure, and there was no obvious saturation platform. During the adsorption process, the interaction between solid surface gas molecules was relatively weak, and the interaction between adsorption molecules was stronger than that between molecules and surfaces. In combination with Figure 2, it can be surmised that the larger the specific surface area of desulfurization dross, the more efficient the physical and chemical adsorption capacity that can be achieved, and the stronger the surface activity and catalytic ability [31,32]. This adsorption characteristic showed that the desulfurization dross had a certain adsorption capacity for small molecular substances in gas (such as SO_2_) or liquid, which can assist the absorption of SO_2_ in wet flue gas desulfurization, or can improve the dewatering performance by adsorbing water during the sludge dewatering process.

### 2.2. Shape Analysis of Desulfurization Dross

**Figure 4:**
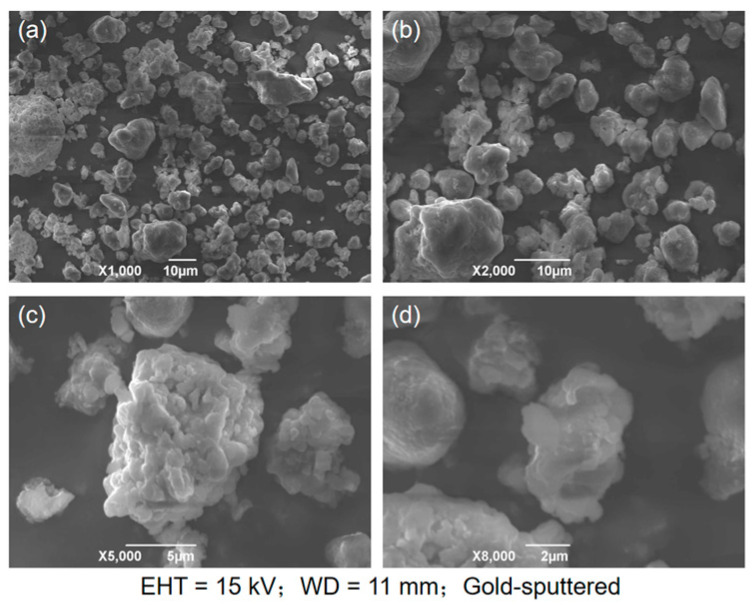
SEM image of desulfurization dross from a Ma’anshan company: (**a**) 1000× times; (**b**) 2000× times; (**c**) 5000× times; (**d**) 8000× times.

The desulfurization dross was detected by scanning electron microscopy (JSM-6490LV, produced by JEOL in Tokyo, Japan), and Figure 4 shows the SEM images of the desulfurization dross ground and sieved to 200 mesh at different magnifications. The coefficient of variation in desulfurization dross particles gradually decreases with increasing magnification, ranging from 0.14 to 0.24. This indicates a higher uniformity in particle morphology at smaller scales. At larger scales (e.g., 1000× times), increased morphological variability is observed, likely due to particle agglomeration or surface attachments.

**Figure 5:**
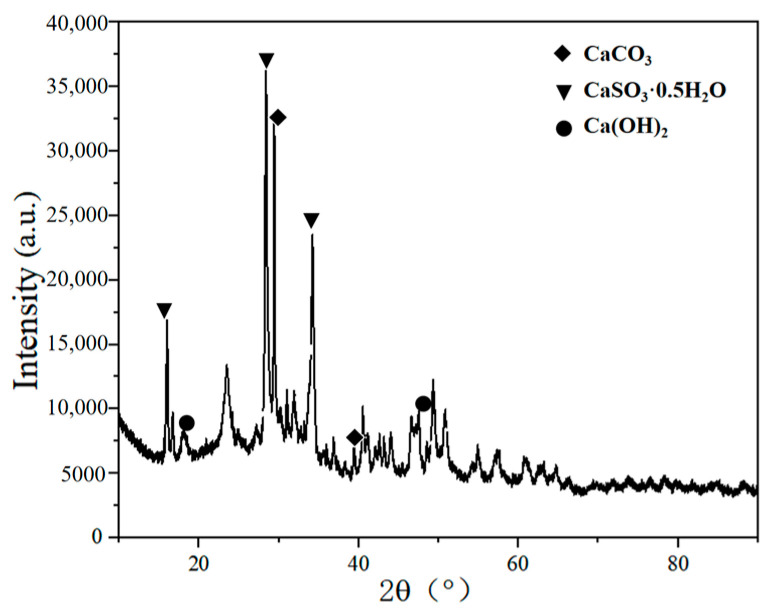
XRD diagram of desulfurization dross from a Ma’anshan company.

From Figure 4, it can be seen that the desulfurization dross particles were irregular in shape, porous, and agglomerated. The reasons for the above may be the following: (1) In the process of desulfurization, there were differences in reaction conditions such as temperature, reactant concentration reaction time, etc., in different areas. In areas with high local temperature and a large reaction contact area, the reaction rate was fast and the desulfurization dross particles were larger, and vice versa. (2) The desulfurization reaction was a complex multiphase reaction, and the newly formed desulfurization product was gradually deposited on the original particles. Due to the difference in reaction sites and reaction rates, the surface growth was uneven, forming an irregular shape. (3) It was difficult for desulfurization products to form a liquid phase, and even if a significant solid-phase diffusion effect could be produced, it was impossible to achieve strong densification, so the surface structure of semi-dry desulfurization dross was loose and porous [33]. (4) Under the action of surface tension, the surface energy reached a minimum, resulting in the desulfurization dross particles becoming spherical. (5) There was a charge on the surface of the desulfurization dross particles, and particles with opposite charges attracted each other, in agglomeration. The particles of semi-dry desulfurization dross were mostly close to spherical, with a relatively dense structure, rougher surface, and stronger cohesion. During the desulfurization process, the surface of these desulfurization dross particles may adsorb desulfurization products, and then form a dense structure, which impedes the continuation of the desulfurization reaction, leaving effective desulfurization substances inside. Therefore, the semi-dry desulfurization dross can be recycled for wet flue gas desulfurization [32,33,34]. The desulfurization dross sample was ground prior to analysis to eliminate interference from factors such as particle agglomeration and an uneven size distribution, thereby ensuring a clear observation of the microscopic morphology and surface structure of individual particles. The desulfurization dross detected by Zhang et al. [25] is shown in Figure 5, and it can be found that the microscopic morphology of different semi-dry desulfurization dross particles was generally the same, which was spherical with an irregular surface.

The desulfurization dross of the semi-dry method had the characteristics of a fine particle size, loose and porous surface, large specific surface area, etc., and the reaction activity was better. The desulfurization dross can be reused better after pulverizing the particles [35,36].

### 2.3. Component Analysis of Desulfurization Dross Provided by a Company in Ma’anshan

**Figure 6:**
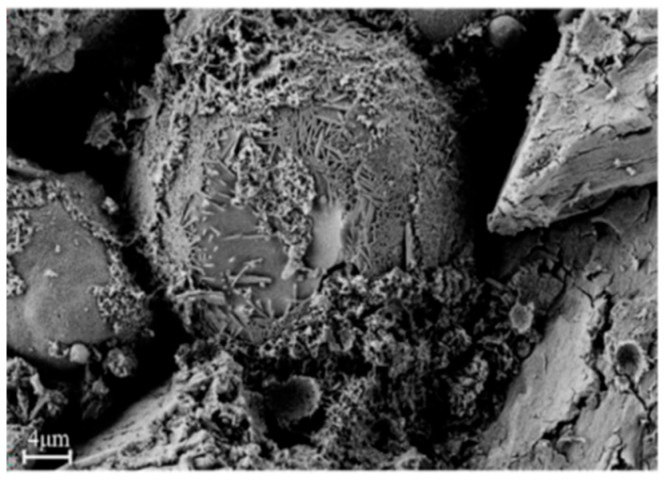
SEM image of lightweight soil after curing for 7 d [40].

The component of desulfurization dross from the semi-dry method was analyzed by X-ray diffractometer (D8ADVANCE, produced by Bruker in Karlsruhe, Germany) and X-ray fluorescence spectrometer (X’Pert PRO, produced by PANalytical in Almelo, The Netherlands), and the two testing results corroborated each other. According to the results of XRD and XRF tests, it can be judged whether the desulfurization dross belongs to high-sulfur and high-calcium desulfurization dross. According to the characteristics of the composition, the desulfurization dross from the semi-dry method can be reused [17]. The-ray diffraction instrument (XRD) was used to detect the desulfurization dross, and the detection results are shown in Figure 6. An X-ray fluorescence spectrometer (XRF) was used to detect the components of the desulfurization dross, and those are shown in Table 3.

It can be seen from Figure 6 that the mineral phase of the desulfurization dross was mainly composed of CaSO_3_∙0.5H_2_O, CaCO_3_, and Ca(OH)_2_.

It can be seen from Table 2 that the main components in the desulfurization dross were CaO, SO_3_, Cl, Fe_2_O_3_, K_2_O, MgO, SiO_2_, TiO_2_, Na_2_O, and Al_2_O_3_, in addition to other metal oxides. Of them, the most abundant was CaO, which accounted for 55.49%. SO_3_ was the second most abundant, with a content of 31.3%, indicating that the self-tested desulfurization dross belonged to a typical high-calcium and high-sulfur complex compound. Chen et al. [17] tested three types of desulfurization dross and concluded that they belonged to a high-calcium and high-sulfur complex compound. In Figure 3, it can be seen that calcium oxide in the desulfurization dross mainly existed in the form of CaSO_3_∙0.5H_2_O. The semi-dry desulfurization dross also contained small amounts of Cl, Fe, Si, and other elements, which will harm its utilization value in agriculture, construction, and other fields if directly buried [25]. During the desulfurization process, the calcium-based desulfurizer reacted with sulfur dioxide in the sintering flue gas, eventually generating calcium sulfite. If the calcium-based desulfurizer was excessive during the desulfurization process or the reaction was incomplete, there may have been an amount of calcium carbonate and calcium hydroxide remaining in the desulfurization dross. It can be seen from Figure 6 and Table 3 that the desulfurization dross submitted for inspection was not completely desulfurized. In addition to the product of the desulfurization reaction CaSO_3_∙0.5H_2_O, there was still unreacted limestone (CaCO_3_), Ca(OH)_2_, and other substances in the desulfurization dross. The desulfurization dross also contained impurities generated due to the reaction of impurities in the desulfurizing agent or other components in the flue gas. The main elements when preparing sulfo-aluminate cement clinker are Ca, S, O, Si, etc. [37]. According to the composition of the semi-dry desulfurization dross, it can be used as the main raw production material for sulfo-aluminate cement.

## 3. Resource Utilization for Semi-Dry Desulfurization Dross

The semi-dry desulfurization dross was high-calcium and high-sulfur, with a fine particle size, porous surface desulfurization dross particles, and a large specific surface area. Because of these characteristics, the semi-dry desulfurization dross can be used as binding for building materials; because of the high content of Ca(OH)_2_ in the desulfurization dross, its alkalinity makes it suitable for ameliorating acidic soil; the desulfurization dross also contained residual desulfurizer, which can be used instead of part of the desulfurizer for wet desulfurization; due to the porosity of the desulfurization dross, it can adsorb and purify sludge wastewater; and the desulfurization dross contained a large amount of CaSO_3_∙0.5H_2_O, CaCO_3_, Ca(OH)_2_, and CaO, which can be used for high-value utilization of the desulfurization dross. Therefore, this research on the resource utilization of semi-dry desulfurization gypsum mainly focused on building materials, agriculture, wet desulfurization, sludge wastewater treatment, and high-value utilization in other fields.

### 3.1. Building Material Field

The construction materials sector represents the largest and most technologically mature avenue for the utilization of semi-dry desulfurization dross. When leveraging its cementitious properties, filling capacity, and alkalinity, it can partially replace natural raw materials or industrial additives. Pretreatment processes are employed to address compositional instability, ensuring compatibility with the performance requirements of various building materials. Zhu et al. [23] found that when using deionized water to simulate natural leaching of desulfurization dross, the leached heavy metal concentrations were as follows: Cu^2+^ ≤ 0.021 mg/L, Zn^2+^ ≤ 0.180 mg/L, Pb^2+^ ≤ 0.494 mg/L, Cd^2+^ ≤ 0.070 mg/L. All values were below the limits specified in the Identification Standards for Hazardous Wastes, indicating that the material does not qualify as hazardous waste. This provides a critical environmental safety basis for its resource utilization.

In road engineering applications, the cementitious properties and economic advantages of desulfurization dross are particularly significant. The semi-dry desulfurization dross can be used for road filling backfill because of its characteristics: (1) the desulfurization dross can contribute to cementitious reactions similar to cement, generating calcium-silicate hydrate and forming strength, so it can be used as a high-quality road base material; and (2) the desulfurization dross, as an industrial combustion waste residue, is low-priced, at only one-tenth the price of market expanders, and using it as road filling backfill for recycling can not only reduce engineering costs but also reduce the waste of mineral resources. Liu et al. [38] applied desulfurization dross to the base course of road cement-stabilized crushed stone, using the expansion performance of desulfurization dross itself to partially replace the expansion agent. Gao et al. [39] found that the use of desulfurization slag to partially or fully replace mineral powder as the fine filler of an asphalt mixture had high feasibility. When the particle size was the same as that of the mineral powder, desulfurization slag could replace the mineral powder, and the desulfurization slag with a high calcium content had a favorable effect on the adhesion performance of asphalt. Currently, the market price of slag micropowder ranges from 200 to 300 CNY/t, while desulfurization dross can be acquired at a minimum cost of 20 CNY/t [18]. By incorporating oxidatively modified desulfurization dross into slag micropowder applications, not only can it meet substantial market demand but it also it can add significant value, generating considerable economic benefits for enterprises. Zuo et al. [40] prepared lightweight soil for road backfill with desulfurization slag and fly dross as raw materials. Through the application of desulfurization dross to the disintegration of fly dross, the desulfurization slag particles were partially wrapped in flocculent and needle-like substances over 7 days of curing, which enhanced the interlocking force between particles and the formation of cementitious materials, thus further improving the strength. The SEM chart of lightweight soil solidified for 7 days of curing is shown in Figure 6.

Zhang et al. [41] developed a desulfurization slag-based soil solidifier and found that as the dosage of desulfurization slag increased, the setting time of the desulfurization slag-based soil solidifier showed an increasing trend and the mortar strength showed a decreasing trend, meaning the addition of desulfurization dross should be less than or equal to 30%. Desulfurization dross can also partially replace cement in cement production. This approach not only enables the efficient utilization of industrial solid waste and reduces environmental pollution but also enhances corporate economic benefits, achieving a win–win outcome for both environmental protection and economic development. It provides an effective solution for waste utilization and pollution control. Compared with traditional cement mortar, it had advantages such as crack resistance, no hollowing, and good thermal insulation [42]. Desulfurization dross had a large recycling value because it was often used as raw materials for cement production due to its cementitious properties [26]. Zhong et al. [43] utilized desulfurization dross to replace a portion of cement raw materials, which can reduce clinker burning energy consumption. When the sulfur content in clinker increased by 0.14%, the 28-day strength increased by 2 MPa. The process of adding desulfurization dross to Granulated blast furnace slag powder and then mixing it into cement is mature and stable. Su et al. [44] added 1–3% desulfurization dross to ground and dried slag micropowder and conducted repeatability tests every 2 h to determine the chloride ion content in ground, granulated blast furnace slag powder with different blending ratios. As shown in the accompanying Table 4, the repeatability verification demonstrated that the chemical composition of the slag micropowder remained stable with the incorporation of 1–2% desulfurization dross, with no significant adverse effects on its physicochemical properties. The 28-day activity index exceeded 95%, and all measured indicators complied with the requirements specified in the Chinese National Standard GB/T 18046-2017 for slag micropowder [45]. The mechanical strength of the mixtures without desulfurization dross and with desulfurization dross is shown in Table 4, which includes three sets of data: Group A (without desulfurization dross added), Group B (with 12% desulfurization dross added), and Group C (with 11.25% desulfurization dross added).

The desulfurization dross contained a large amount of f-CaO and SO_3_^2–^, which can enhance the bonding performance, and it can be used as a binder and mixed with cement [46]. Wu et al. [26] found that the high content of CaO in the desulfurization dross could effectively activate the activity of mineral powder, promote its hydration, and produce certain strength, which could reach 70% that of cementitious materials. Carro-López et al. [47] found that the compressive strength of cement mortar with a ratio of desulfurization dross to cement of 2:3 exceeded 52.5 MPa at 28 d. Chi et al. [48] found that desulfurization dross can replace part of the cement or can be used as a substitute for fly dross, but in ordinary Portland cement, its addition amount needs to be controlled within 20%. Li et al. [49] found that when the addition of superfine desulfurization dross did not exceed 30%, the mixture of this cement and superfine desulfurization dross had better rheological properties than pure cement paste. Shi et al. [50] used desulfurization dross to activate mineral powder to prepare modified admixtures, and its 7 d and 28d compressive strength was significantly improved, especially when the addition of desulfurization dross was 10%; its 7 d compressive strength was increased by about 3% compared with the mineral powder-cement mortar group without adding desulfurization dross. Fan et al. [51] improved the strength activity of desulfurization dross residue by mechanical grinding, and they compounded the ground desulfurization dross with fly dross at the ratio of 3:7 to prepare composite mixtures. The composite admixture of desulfurization dross residue can replace 20% of cement, and for the C30 desulfurization dross residue concrete prepared, the 28 d compressive strength reached 41.1 MPa, which was far higher than the requirements for preparing C30 concrete.

Desulfurization dross can also be used in the field of concrete. Yang et al. [52] found that adding an appropriate amount of desulfurization dross to concrete had a filling effect, reduced the proportion of pores inside the matrix, and improved its degree of compaction, thereby improving the compressive strength. The replacement of cement with desulfurization dross can improve the dry shrinkage performance of the mixture, but it will reduce the compressive strength. Compared with the pure cement-stabilized recycled aggregate mixture, the frost resistance of cement desulfurization dross-stabilized recycled aggregate mixture was poor, but it can improve the dry shrinkage performance [53]. Chen et al. [54] found that the incorporation of 30% desulfurization dross could improve the 7 d compressive strength and flexural strength of plaster dross mortar, but Ca(OH)_2_ in the desulfurization dross will shorten the setting time, and it was necessary to select alkali-resistant orders to improve fluidity. An appropriate amount of desulfurization dross was added to the autoclaved aerated concrete, which can not only improve the gasification effect but also enhance the compressive strength to a certain extent. Xu et al. [55] found that autoclaved, aerated concrete mixed with an appropriate amount of sintered desulfurization dross had a higher content of tobermorite and improved crystallinity, which gave it higher strength. The components of desulfurization dross fluctuated greatly, and the unstable calcium sulfite could be improved by pretreatment (such as grinding, oxidation). Cao et al. [30] used semi-dry desulfurization dross after water digestion, which can improve the compressive strength of aerated concrete and reduce its dry density at the same time; the use of ball milling to grind the semi-dry desulfurization dross improved the compressive strength of aerated concrete, reduced its dry density, and reduced the content of CaSO_4_ in the chemical composition, which improved the crystallinity of tobermorite and made its distribution more compact. Based on the concept of multi-source solid waste synergistic utilization, Zhang et al. [56] prepared high-value, lightweight, energy-saving wall materials with semi-dry sintering desulfurization dross, fly dross, and cement as the main raw materials under non-autoclaved curing conditions, achieving a compressive strength of 8.64 MPa at 28 d; this process does not require pre-oxidation treatment of desulfurization dross and provides technical support for its simple and high-value utilization.

**Figure 7:**
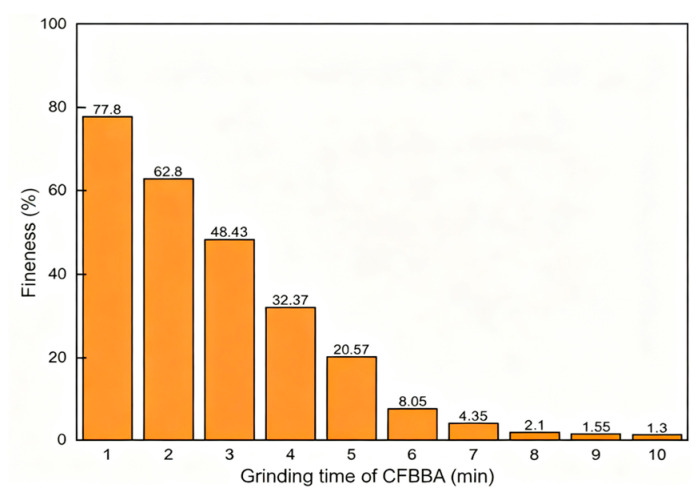
Effect of grinding time on the fineness of CFBBA [57].

The direct resource utilization of semi-dry desulfurization dross is constrained by its chemical instability, primarily due to the potential risks of volume expansion and strength degradation caused by free calcium oxide (f-CaO) and calcium sulfite. Studies have shown that hydration digestion can effectively convert f-CaO into Ca(OH)_2_ in advance, significantly improving the volume stability of the material and avoiding the risk of delayed expansion in products. Mechanical grinding, on the other hand, optimizes the particle size distribution, increases the specific surface area, disrupts dense structures, releases internal active components, and enhances its compactness as a filler in matrices. Additionally, low-temperature and humid storage conditions must be strictly avoided to prevent the formation of non-strengthening thaumasite, which can lead to a deterioration in product performance [55]. Therefore, targeted pretreatment is a crucial preliminary step for achieving efficient and safe utilization. Du et al. [57] used the grinding process to treat CFB method desulfurization dross (CFBBA) in order to produce cement admixture; the fineness of desulfurization dross had met the standard GB/T 1596-2017 [58] after grinding for 5 min, and the effect of grinding time on fineness can be seen in Figure 7.

The effectiveness of semi-dry desulfurization dross in building materials is strongly influenced by the interplay of the pretreatment process, dosage ratio, and type of matrix material. In applications with relatively lenient strength requirements, such as road subgrades or cement raw meal, the allowable incorporation ratio of desulfurization dross can be higher. In contrast, for high-performance materials like autoclaved, aerated concrete or dross plaster, the dosage must be carefully controlled and combined with pretreatment methods such as hydration digestion or mechanical grinding to ensure performance stability. This situation also highlights a current research gap: the absence of a unified “composition–process–performance” correlation model. As a result, parameters must be repeatedly determined through trial experiments for different application scenarios, which hinders large-scale promotion.

### 3.2. Agricultural Field

Soil acidification is a major problem that has hindered the development of agricultural planting in recent years. It is mainly caused by the loss of large alkaline bases such as calcium, magnesium, and potassium due to rainfall leaching, coupled with the lack of traditional agricultural measures and the long-term use of a large amount of chemical fertilizers, which has led to an imbalance of soil nutrients [59]. Soil acidification not only exacerbates soil compaction and reduces fertilizer utilization efficiency but also leads to slow growth of crops, an increase in diseases, and a decrease in crop yield and quality. Phoungthong et al. [60] found that desulfurization dross can be reused as an alternative material for civil applications, but the pollutants contained in the desulfurization dross may pollute the local environment, hindering its material reuse. Before use, the phytotoxicity leachate produced by desulfurization dross can be assessed. However, the harmful heavy metals contained in the desulfurization dross may accumulate in the environment and cause poisoning to animals or humans through the food chain, and they may also indirectly harm animals, plants, and human health through the pollution of groundwater [61]. The semi-desulfurization dross was alkaline and can improve the acidity of the soil, increase the pH value, releases the mineral elements needed by plants, and at the same time, reduced the accumulation of toxic soluble metals in the soil, as well as improved the soil properties. The agricultural sector can utilize the strong alkalinity and nutrient-releasing capacity of semi-dry desulfurization dross for acidic soil amelioration and cultivation substrate preparation. However, it is essential to strictly control heavy metal risks to ensure agricultural environmental safety.

**Figure 8:**
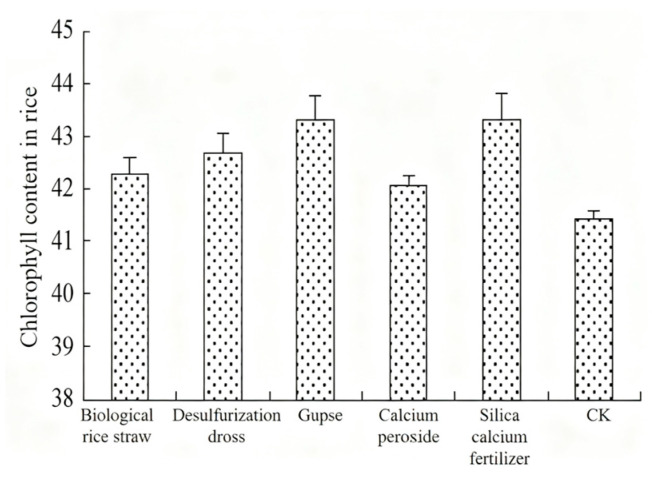
Effects of different regulators on chlorophyll content in rice leaves [63].

Dong et al. [62] found that the application of bioactive carbon and desulfurization dross could enhance the rice wound flow, root–crown ratio, and biomass. Liu et al. [63] found through field trials that the application of desulfurization dross as a soil conditioner in cold paddies significantly increased the effective phosphorus and effective potassium of the soil, and at the same time, increased the chlorophyll content of rice leaves by 3%. Moreover, the use of desulfurization dross in agriculture was less expensive than other conditioners such as dross. The effects of different conditioners on the chlorophyll content of leaves are shown in Figure 8.

Zhou et al. [64] used fly dross and desulfurization dross as the main raw materials to prepare non-fired expanded perlite through steam curing. The perlite had a developed internal pore structure and a large water absorption rate, and it was rich in elements such as Si, Ca, and S, meaning it was suitable for plant growth needs; the non-fired planting expanded perlite was a colloidal aluminosilicate network-structure geopolymer binder, which had a suitable structure and strong durability.

### 3.3. Wet Flue Gas Desulfurization

As a by-product of desulfurization, the semi-dry desulfurization dross contained a certain amount of effective desulfurization components. By adopting a “waste-treats-waste” approach, the residual active desulfurization components in the dross can be utilized to partially replace limestone-based desulfurizing agents, thereby reducing desulfurization costs while enabling the cyclic utilization of the desulfurization dross. Li et al. [65] found that it was feasible to partially replace limestone powder with calcium-based desulfurization dross in wet flue gas desulfurization systems. While pure limestone powder achieved a desulfurization efficiency of 99%, when the substitution ratio of desulfurization dross was controlled within 50%, the desulfurization efficiency of the system could be maintained above 95%. Yang et al. [66] used semi-dry desulfurization dross in wet flue gas desulfurization, and the desulfurization efficiency decreased with the increase in flue gas temperature, flow rate, and SO_2_ concentration, while it increased with the increase in liquid–gas ratio and pH; and compared with the traditional wet flue gas desulfurization agent limestone, the difference in desulfurization efficiency was no more than 5%, while the dry desulfurization dross had a desulfurization efficiency of more than 90% and strong desulfurization activity, which could meet the emission index of desulfurization efficiency, at no less than 95%. For wet flue gas desulfurization, semi-dry desulfurization will face problems such as a poor desulfurization efficiency, small desulfurization capacity, and high slurry concentration [65]. Zhang et al. [25] found that when the mixing ratio of coke oven desulfurization dross and semi-dry desulfurization dross was 5%, the desulfurization effect was significantly improved, and the average desulfurization efficiency could be maintained above 96% under the conditions of a low temperature, high SO_2_ concentration, and fast flue gas flow rate, while the maximum could reach 99%, which greatly improved the desulfurization efficiency of semi-dry desulfurization dross. The application of semi-dry desulfurization dross in wet flue gas desulfurization systems requires a comprehensive consideration of process compatibility, operational stability, and potential risks. In practice, excessively large particle sizes in the dross may lead to clogging of spray nozzles in the absorption tower, reducing desulfurization efficiency. Meanwhile, a high Cl^−^ content can accelerate equipment corrosion and shorten the service life. By optimizing pretreatment processes—such as grinding for particle size reduction and drossing for chloride removal—the economic and environmental benefits of its utilization can be fully realized.

**Figure 9:**
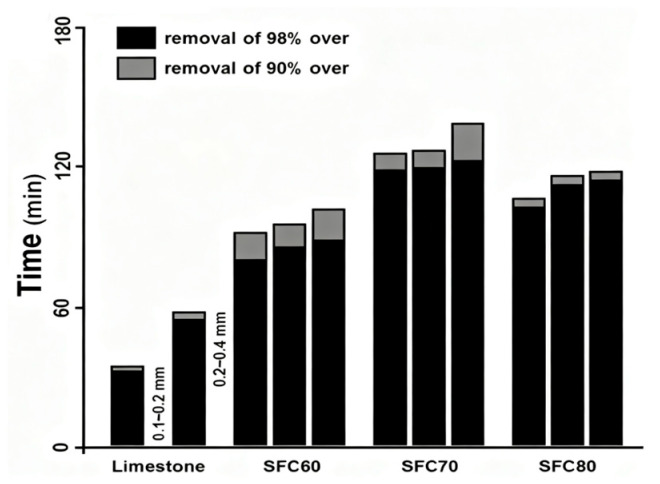
Absorption time measurements of Ca-based desulfurization sorbents [69].

The content of CaSO_3_ was one of the factors affecting the dissolution rate of desulfurization dross, and the higher the content of CaSO_3_, the lower the dissolution rate. The semi-dry desulfurization dross was alkaline due to the presence of a large amount of CaO and Ca(OH)_2_. It can partially replace limestone for wet flue gas desulfurization, though its dissolution characteristics affect the desulfurization performance. Jia et al. [67] found that reducing the pH value of the solution and appropriately increasing the proportion of desulfurization dross addition were beneficial for the dissolution of the desulfurization agent. Due to the relatively poor oxidation conditions of the semi-dry desulfurization dross, the content of calcium sulfite in the desulfurization dross higher. Wang et al. [68] conducted an experiment that involved adding desulfurization dross to limestone for wet desulfurization, and they found that the addition of 25% desulfurization dross could effectively oxidize calcium sulfite in the desulfurization dross. Baek et al. [69] tested the SO_2_ concentration of a fluidized bed reactor filled with limestone and calcium-based desulfurization adsorbents (SFC60, SFC70, and FC80), and the time for a desulfurization rate of 90% was less than 2 h, while the results of the adsorption time measurement of calcium-based desulfurization adsorbents are shown in Figure 9. As an industrial by-product, desulfurization dross is generated in large quantities and boasts raw material costs significantly lower than those of traditional coagulants. Utilizing desulfurization dross in the production of coagulants can also reduce landfill disposal expenses, further amplifying its economic advantages.

Studies indicate that the core value of semi-dry desulfurization dross in sludge and wastewater treatment lies in its multi-functional synergy: in sludge treatment, it simultaneously provides skeletal support, moisture adsorption, and structural conditioning; in wastewater treatment, it offers alkaline adjustment, coagulation, and heavy metal adsorption capabilities. Sulfuric acid modification has been identified as a key pretreatment method for enhancing wastewater treatment efficiency, thus significantly improving turbidity and heavy metal removal rates. While current research has covered applications in municipal sludge and mine wastewater, its effectiveness in treating complex effluents such as high-salinity wastewater and dyeing wastewater remains unexplored, indicating a need for broader application scenarios.

### 3.4. Treatment for Sludge and Wastewater

When leveraging the porous adsorption capacity, alkalinity regulation ability, and skeletal support function of semi-dry desulfurization dross, it can be applied in sludge and wastewater treatment. This approach addresses challenges such as the poor sludge dewaterability and low removal efficiency of pollutants in wastewater, enabling the synergistic treatment of solid waste and wastewater. Sludge from cities has the characteristics of large production, a high water content, a complex composition, difficult dewatering, and a large amount of plant nutrients. The treatment cost was relatively high, and dewatering the sludge at a low cost became the key to sludge treatment. Oxidation by potassium permanganate, skeleton construction by desulfurized dross, and an improvement in sludge dewatering performance can be achieved by strengthening the thermal effect and chemical oxidation effect of the combined technology of microwaves, ultrasonic waves, and ultraviolet rays (PUWU) in turn [70,71,72]. Tai et al. [72] found that after coupling treatment with PUWU, potassium ferrate and desulfurization dross, the sludge structure became denser, a continuous morphology was formed on the surface, the porosity was reduced, and the solid particles were arranged closely, which helped the filter of water. Ouyang et al. [73] used desulfurization dross and oily sludge to prepare pellets. After high-temperature firing, pellets had a good solidification effect on heavy metals such as Cd, Cr, Pb, and Zn, with a large density and low water absorption.

The desulfurization dross modified by sulfuric acid will form a complex with a complex structure and composition, which can reduce the surface potential of suspended particles in water, weaken the same-charge repulsion between charged particles, and promote the collision and agglomeration of particles to form flocs, so that the particles in water can be precipitated. Therefore, it can be used as a coagulant to treat wastewater [74]. Jiu et al. [75] modified the desulfurization dross from coal-fired power plants with sulfuric acid, and the research showed that the desulfurization dross after modification with sulfuric acid had a good performance in removing turbidity from mine wastewater. Fang et al. [76] utilized semi-dry desulfurization dross to remove chromium and vanadium from wastewater, and the residual mass concentrations of Cr(VI), total Cr, and V were 0.63 mg·L^−1^, 0.395 mg·L^−1^, and 0.155 mg·L^−1^, respectively.

### 3.5. High-Value-Added Utilization

High-value-added utilization is a crucial direction for upgrading semi-dry desulfurization dross from low-value consumption to high-value conversion. Through chemical transformation or modification, it can be processed into high-purity and highly functional products, thereby enhancing its economic value. Zeng et al. [77] successfully prepared high-purity calcium sulfate powder by reacting 70 g of desulfurization dross, 300 mL of water, and 45 g of hydrogen peroxide for 90 min, and then adding 550 g of dilute sulfuric for 60 min. The conversion rate of calcium sulfite was 97.86%, and the content of calcium sulfate in the product was 98.4%.

Zhang et al. [78] modified the desulfurization dross through chemical modification treatment, and they used the modified desulfurization dross to replace part of the carbon black to prepare modified desulfurization dross-based ecological rubber; the preparation process was analyzed by XRD, SEM, etc. It was found after the modified desulfurization dross was added, the maximum torque of the ecological rubber compound decreased significantly, and the scorching time and the normal vulcanization time were. The vulcanization process was divided into the induction period, the reaction period, and the flat period, and different cross-linked network structures were formed in each stage. Lu et al. [79] used desulfurization dross as reinforcing filler and prepared a natural rubber (NR)/desulfurization dross composite by direct mixing, which shortened the scorching period of NR and improved the efficiency of vulcanization, and it also improved the thermal stability and mechanical properties of the composite, as well as improved the comprehensiveness of NR in a low-cost manner. Lv et al. [80] used a boiling furnace to catalytically decompose desulfurization dross with a composite catalyst composed ferrous sulfide and ferrous sulfate at a reaction temperature of 900 °C, achieving a desulfurization rate of 91.41% The generated SO_2_ can be used to produce sulfuric acid, and the roasting product can be used as a raw material for sintering plants. Mao et al. [81] reduced and calcined the sinter SDA desulfurization dross, and they analyzed SO_2_ for acid production, finding that the solid product can be recycled.

Du et al. [82] used desulfurized dross as a catalyst for the pyrolysis of cotton stalk at 900 °C and found that the desulfurized dross could promote the pyrolysis of cotton stalk, improve the yield of pyrolysis gas and the low calorific value, promote the generation of H_2_ from cellulose and CH_4_ from lignin, and inhibit the generation of CO and CO_2_ from cellulose.

In terms of industrial scalability, most current research on high-value applications remains at the laboratory or pilot stage, and transitioning to a large-scale industrial implementation still faces multiple bottlenecks. The variability in the composition of desulfurization dross from different production sources poses a significant challenge to chemical conversion processes, which require highly stable input materials.

### 3.6. Environmental Risk

The environmental risks associated with semi-dry calcium-based desulfurization dross stem from its complex physicochemical properties. During its reuse in agriculture, construction, and other fields, these risks may be realized through various pathways of toxicity, necessitating targeted identification and management.

Although the construction sector mitigates risks through solidification, long-term use or improper disposal still poses potential hazards. When used in wall materials, desulfurization dross must undergo sufficient pretreatment to meet the particle size distribution and harmful substance content requirements specified in Recycled Coarse Aggregate for Concrete (GB/T 25177-2010) [83]. The Technical Guidelines for Construction of Highway Roadbase Bases (JTG/T 3650-2020) require that the pH of grouting materials be between 6.5 and 8.0 [84]. Therefore, when desulfurization dross is used in road base materials, its dosage must be controlled to avoid strong alkalinity causing corrosion to the pavement.

In agricultural applications, desulfurization dross may leach heavy metals such as Pb, Ni, and Cd. Testing should be conducted according to the methods specified in Fertilizers and Soil Conditioners: Determination of Arsenic, Cadmium, Chromium, Lead, and Mercury Contents (BS ISO 17318:2015) [23,25,85,86]. If heavy metal leaching concentrations are excessively high, these metals can migrate through the “soil–crop root absorption–plant accumulation” pathway. The accumulation of heavy metals in crops may exceed the limits set in the National Food Safety Standard: Limits of Contaminants in Food, ultimately endangering human health through the food chain [23,25,87]. Moreover, current research predominantly focuses on the short-term effects of desulfurization dross on crop growth, while long-term data tracking its impact on soil microbial communities and the evolution of soil physicochemical properties remain scarce. Addressing this knowledge gap represents a critical direction for future studies.

In addition to conventional uses, the high-value extraction and targeted conversion of calcium resources from this dross align with the core principles of the circular economy—reduce, reuse, recycle—and can generate significant economic and environmental benefits. Although technical and economic challenges remain, increasingly stringent environmental policies, rising resource costs, and carbon neutrality goals provide a strong impetus for innovation. These emerging pathways hold considerable potential and long-term significance, enabling a genuine integration of environmental and economic benefits.

## 4. Conclusions and Outlook

(1) The resource utilization of semi-dry calcium-based desulfurization dross is an important direction to promote the treatment of industrial solid waste and the development of a circular economy. Desulfurization dross had shown the feasibility of replacing natural materials in building materials, agriculture, and other fields, such as reducing cement energy consumption and improving acidic soil, and its economy was better than traditional methods. Desulfurization dross can also be used for wet desulfurization, wastewater treatment, and high-value-added utilization, which can achieve “waste treatment”.

(2) The primary technical challenge stems from the highly volatile composition of materials, which leads to an inconsistent product performance. Existing pretreatment processes are constrained by high costs and energy consumption, making them economically unviable. There is an urgent need to develop scalable pretreatment technologies. On the regulatory front, the absence of targeted product standards, environmental access regulations, and comprehensive risk management systems prevents the effective assessment of long-term environmental impacts and ecological accumulation effects, thereby increasing uncertainties in large-scale implementation.

(3) To systematically advance development in this field, we should establish long-term environmental behavior monitoring and risk assessment models based on real-world application scenarios; accelerate the formulation of classification standards, usage specifications, and environmental release thresholds for desulfurization dross-based products to build a comprehensive standard system; and develop a “cascade utilization” model for desulfurization dross: initially, replace limestone in wet flue gas desulfurization processes, and then repurpose the resulting secondary desulfurization dross as a construction filler to enhance resource efficiency. Only through coordinated efforts in technological innovation, standard refinement, and regulatory mechanism innovation can we break current bottlenecks and achieve high-value, large-scale, and environmentally safe utilization of semi-dry desulfurization dross.

## Figures and Tables

**Table 1:** Classification of typical calcium-based desulfurization dross in iron and steel industry.

Classification of Desulfurization Dross	Wet Flue Gas Desulfurization Dross	Semi-Dry Desulfurization Dross	Dry Desulfurization Dross
Desulfurization process source	Lime-dross method	CFB; SDA	Activated carbon adsorption; dry shot blasting; dense-phase tower (DFA)

**Table 2:** Comparison of SDA and DFA methods [18,19].

Comparison Dimension	SDA	DFA
Key Equipment	Lime slurry is atomized and contacts flue gas in parallel flow; after reaction, dry desulfurization dross is formed through drying	Powdered Ca(OH)_2_ desulfurizer is fully mixed with flue gas in countercurrent flow in a dense-phase tower. The reaction is enhanced by means of a high-concentration bed
Process Limitations	Rotary atomizer	Dense-phase reaction tower + high-efficiency dust collector
Process Limitations	Wear of the atomizer leads to high operating costs	Local wall sticking is prone to occur in the dense-phase bed, requiring control of wind speed and bed concentration

**Table 3:** Composition of desulfurization dross.

Chemical Composition	CaO	SO_3_	Cl	Fe_2_O_3_	K_2_O	MgO	SiO_2_	TiO_2_	Na_2_O	Al_2_O_3_	Others
Content (%)	55.49	31.33	4.10	3.47	1.82	0.87	0.82	0.59	0.51	0.49	0.51
Standard error	0.25	0.23	0.10	0.09	0.07	0.043	0.041	0.03	0.025	0.025	0.025

**Table 4:** Composition of desulfurization dross [44].

Group	A	B	C
Compressive Strength Before Freeze–thaw Cycles/MPa	6.3	5.4	6.6
Tensile Strength	0.42	0.30	0.40
Compressive Strength After Freeze–thaw Cycles (5 Freeze–thaw Cycles)	5.8	-	5.6

## Data Availability

No new data were created or analyzed in this study. Data sharing is not applicable to this article.
